# Custodian for elderly with memory impairment in Sweden – a study of 260 physicians’ statements to the court

**DOI:** 10.1186/1471-2318-14-94

**Published:** 2014-08-26

**Authors:** Karin S Björkstén, Kerstin Fälldin, Johanna Ulfvarson

**Affiliations:** 1 Psychiatry South Stockholm, Ledning & Administration, Box 5040, SE-121 05 Johanneshov, Sweden; 2 Department of Neurobiology, Care Sciences and Society, Karolinska Institutet, Stockholm, Sweden; 3 Chief Guardian’s Office of the City of Stockholm, Stockholm, Sweden

**Keywords:** Mental capacity, Physician’s statement, Court, Custodian, Capacity assessment, Memory impairment, Dementia, Elderly

## Abstract

**Background:**

In the modern world with new family structures, international migration and increased life expectancy, there is a growing need for legal ways of assisting elderly with impaired mental capacity to decide about their life and assets. There are few studies about the physician’s role when a court appoints proxies for vulnerable elderly. Many doctors do not know how to assess mental capacity, and most lawyers and judges know little about medicine.

**Methods:**

Applications for a custodian sent to the Stockholm Chief Guardian’ Office in Sweden were used. Physician’s statements to the court for elderly with memory impairment were selected and 260 statements were scrutinized with regard to formal quality, the narrative content and the physician who wrote it.

**Results:**

The quality of the statements varied from one sentence to excellent. Most statements were written by senior family practitioners or geriatricians. Seventeen % of the statements were handwritten and had more formal shortcomings than machine/computer written statements.

The majority of patients needed massive help with daily life and economy. Median age was 84 years of age. MMSE score was given in 20% of the cases and varied from 6–27.A diagnosis of dementia was established in 57%. At the time of application, at least 48% were in a hospital or nursing home and at least 27% were in their private home. Only 5% were living with a spouse or a child. In 53% of the cases, the doctor knew the patient, but in 40% of the cases, the identity of the patient was not confirmed. The physician found that 54% were unable to understand the idea of getting a custodian, but out of those very vulnerable elderly, 20% had signed consent and 57% were considered able to be heard in court.

**Conclusions:**

There is a large variation in the quality of physicians’ statements to the court concerning the mental capacity of elderly patients with cognitive impairment. Many statements have serious short-comings, and the system is not safe. There is a strong need for guide-lines, and additional training for all professionals involved.

## Background

Modern life with increased life expectancy, international migration, new family structures and new values highlight the need for legal ways of assisting elderly with impaired mental capacity to decide about their life and assets. Most elderly are perfectly able to decide for themselves, but those with multiple medical disorders, cognitive impairment or dementia often need assistance in decision-making not least economic matters. Many elderly do not have close relatives, and others do not want their children or relatives to decide on their behalf. As a consequence, vulnerable elderly may be easy targets for criminal activities concerning money [1,2], wills [3], consumer fraud [4], and marriage for other reasons than love [5]. Cases of financial elder abuse are, however, rarely prosecuted [6].

The rights of the family and court-appointed proxies differ between countries. The European Dementia Consensus Network reviewed how competence (capacity) assessment in dementia is practised in a number of European countries [7] and found a large variety of legislations and practices. The Mental Capacity Act (MCA) of England and Wales is an example of a modern legislation designed to protect adults with reduced decision-making capacity [8-10]. The different states and territories in Australia [11] and the U.S. have different legislations and practices [12]. Lawyers often take for granted that physicians, at least psychiatrists, know how to assess mental capacity. Many physicians do not know how to do this, and medical schools rarely teach assessment of mental capacity. Methods for assessment of decision-making capacity have been developed for different purposes, like the MacArthur Competence Assessment Tool-Treatment for consent to treatment [13]. The hallmarks of capacity assessment are to check if the patient has ability to understand relevant information and to reason about risks and benefits of potential options and can appreciate the nature of his/her situation and the consequences of his/her choice and last but not least is able to express a choice. Lawyers often believe that a diagnosis of dementia implicates lack of capacity. The progress in research and clinical care in the past decades has totally changed the concept of dementia and cognitive disorders, but this is seldom mirrored by revised legislation.

In spite of the importance of a physician’s assessment and statement when a court appoints proxies for vulnerable elderly, few studies have addressed the role of the physician. It is of utmost importance how the physician assesses and explains the mental capacity of the patient to the court, since this has implications for the patient’s right to make decisions.

In a study of 40 statements for guardianship in persons older than 60 years of age in Texas, U.S.A., almost half of the those were based on "unsupported statements of incompetence" [14]. Another study from Israel, scrutinizing 16 statement concluded that the courts accepted documents with extensive shortcomings [15].

### Swedish legislation

In Sweden, there is a well-established system for courts to appoint proxies for elderly who no longer are able to care for their assets. Although the details are local for each country or state, the over-all idea of appointing proxies for persons with reduced decision-making capacity is general. The previous process of declaring adults legally incompetent and appointing guardians was discontinued in 1989 in Sweden, and replaced by three alternatives: The least intrusive is to write a power of attorney to a relative or a friend, the second to have the court appoint a *custodian* (in Swedish a "god man" meaning a "good man") and the third and most intrusive is to have the court appoint a *trustee* (in Swedish a "förvaltare") Any lesser intruding arrangement, such as a power of attorney should be considered before applying for a court-appointed proxy. Guardians are only appointed for minors. The legal framework is found in the Children and Parents Code [16].

### Custodian

The need for a proxy is often first recognized in the medical care system, which often has to initiate an application for a custodian on the patient’s behalf. The court can appoint a custodian to act on another adult’s behalf due to "disease, a weakened state of health, mental disorder or similar condition". The appointment can include 1–3 of the following: to "safeguard his/her rights and/or manage his/her property and/or ensure his/her personal needs". Having a custodian has no effect on the patient’s power to perform legal acts, such as getting married, voting, signing contracts, buying or selling at unreasonable prices, and does thus not protect the patient from being exploited by criminals or greedy relatives. The custodian, who is a layman, and can be a relative or an un-unrelated volunteer and can never decide against the person’s will. If the patient is able to understand, he or she must sign consent. A physician’s statement and a social investigation done by a social worker must be presented to the court. The local Chief Guardian’ Office usually prepares applications to be presented to the court, and supervises guardians, trustees and custodians. Many custodians fulfil their duties in an excellent way, but there is extensive criticism that the system is not secure from several points of view. One of them is the physician’s statements.

### Trustee

If an adult acts highly un-appropriately with money or other assets due to disease, or is weak and cannot resists exploitation from others, it is possible to have a trustee appointed by the court. This is used when everything else has failed. Having a trustee removes the person’s power to perform legal acts within the scope of the trustee’s assignment. As opposed to having a custodian, the trustee can decide against the patient’s will, but can, however, never decide about medical treatment or moving the patient from home to an institution. A person who has a trustee can still vote.

The aim of this work was, on the basis of physician’s statement to the court, to

– describe the elderly patients with memory impairment who apply to the court for a custodian in Sweden

– investigate how the physicians assessed and described mental capacity

– assess who the physicians were with regard to medical speciality, work-place and seniority

– assess the formal quality of the statements

## Methods

### The physician’s statement

The physician responsible for the assessment must be licenced to practice in Sweden, but there are no requirements about additional training. A form established by the National Board of Health and Welfare (Additional file 1) must be used for the physician’s statement, but there are neither medical nor legal guidelines to help the physician write it. The native English speaking reader may believe that the translation of the form is poor, but we can assure that the language is awkward in the original Swedish version as well. An authorized translator failed to translate is so the authors did it together. The form contains boxes to be filled in with formal data about the patient like date of birth and the physician’s work-place. Firstly the physician must write a narrative text to explain the patient’s state of health and describe the conditions that cause the need for a custodian. Then, there are boxes to be ticked "yes" or "no" before the court proceedings. The most important task for the physician is to assess whether the patient understands the idea of appointing a custodian. Finally the physician must conclude what type of help the patient needs, and on what basis by filling in boxes.

### Sample

All applications for custodian or trustee registered at the Stockholm Chief Guardian’ Office from June 2007 to July 2008 were used (n = 1438). Every other consecutive application was selected (n = 719). All applications for a) persons younger than 50 years of age, b) applications for a trustee, c) sent to the wrong municipality or d) lacking a physician’s statement were excluded from this analysis. In 17 cases (2%), the files were not found. The physician’s statements of the remaining 454 applications were scrutinized for every indication of memory impairment. No memory problems were mentioned in 194 cases. In 260 of the cases, either memory related diagnoses like "Alzheimer’s disease" or "dementia" or vague expressions like "slight memory impairment" or "cognitive problems" were mentioned. The physician’s statements of those 260 applications were further examined. The statements were systematically scrutinized by one of the authors (K Björkstén) and a specially trained nurse first separately, and then together. The original version of the statement was used and not data added later by staff at the Chief Guardian’ Office after contacting the physician for additional information. We also checked whether there was a consent signed by the patient.

Basic descriptive statistics were used.

### Ethics

This study has been performed in accordance with the Declaration of Helsinki and was approved by the Regional Ethical Review Board in Stockholm #2009/1428-31/5, and by the Stockholm Chief Guardian.

## Results

### The patients and why they needed a custodian

Demographic and medical data are summarized in Table 1. There were a total of 260 patients aged 51–105 years (Median 84: Mean 81.7 ± 9.1 SD). Two thirds were women (n = 173) aged 51–105 years (Median 85; Mean 83.4 ± 8.9 SD) and one third (n = 87) were men aged 60–100 years (Median 79; Mean 78.3 ± 8.5).

**Table 1:** Demographic and medical data about the patients

**Demographic and medical data**			**N**	**%**
	Median Age years	Range years		100%
All	84	51-105	260	
Women	85	51-105	173	67%
Men	79	60-100	87	33%
Living alone in a private home			107	41%
Living with a spouse or a child			13	5%
Living in a nursing home			68	26%
Homeless			1	
No information about actual living condition			71	27%
**The cause for the need of a custodian as ticked by the doctor**				
Disease			231	89%
Weakened health			70	27%
Mental disorder			25	10%
Similar conditions			14	5%
**Diagnosis according to the narrative text**				
Dementia			149	57%
Unspecific cognitive impairment or memory problems			98	38%
Stroke			31	12%
Other memory-related diagnosis			14	5%
**Duration of memory impairment**				
no information about duration			146	56%
at least three years			50	19%
Between one and three years			42	16%
less than a year			22	8%
**Additional diagnoses mentioned**			181	71%
**Additional diagnoses actively denied**			4	1.5%

Forty-one per cent of the subjects lived alone in their private home and only 5% were living with a spouse or a child. One woman was homeless. There were 26% living in nursing homes, and in the remaining 27% the living conditions were not clarified.

Unpaid bills were mentioned in 22% and collection of debts in 5%. Threat of eviction was mentioned in 2%, and serious disagreement among family members in 2%. In one case, it seemed obvious that the patient would need an trustee instead of a custodian due to the severity of circumstances.

At the time of assessment, 19% were hospitalized, 29% were staying in a nursing home, and 27% in their private home. In 25% of the cases, it was not clear where the patient was.

### Memory workup and diagnosis

Memory workup was started in 75% and was finished in 56%, but was usually not described in any detail. No information about the duration of memory impairment was given in 56% of the cases. The duration was more than three years in 19%, and 1–3 years in 16% of the cases. The memory impairment had lasted less than a year in 8%.

A diagnosis of dementia was explicitly stated in 57% and in 38% more or less vague descriptions of memory impairment. Other specified memory-related diagnoses were present in 5%, and stroke was mentioned in 12%. Communication impairments like aphasia were mentioned in 9%, hearing problems in 6% and visual problems in 7%. A different first language than Swedish was mentioned in 3%.

In 20% (n = 52), the Mini-Mental State Examination (MMSE) or a neuropsychological (n = 16) test had been carried out during the past year. Six patients had done both the MMSE and a neuropsychological testing. MMSE score varied from 6–27, and the mean was 18 ± 5/30 (n = 36). In 6 cases, the score was not given.

### The physicians, their work-places and formal quality of the statements

Since there are no specially appointed physicians, the clinician responsible for the patient writes the statement to the court. The statements were written by physicians working in family practice in 43%, geriatric clinics in 35% and nursing homes in 10%. The physician’s work-place was missing in 7%. A family doctor or a geriatrician may be responsible for a nursing home. Most statements were signed by senior physicians in geriatrics (38%) or family medicine (37%). In 23%, the physicians had signed the statements without indicating specialist status or seniority.

Almost all statements (98%) were written using the correct form, and the physicians had mostly ticked boxes when required by the form. In 83% the statements were machine/computer-written, but in 17%, they were hand-written and more or less legible. Out of the 17% of the statements that were handwritten, 9% lacked work-place and 53% the position of the physician. Forty-one per cent of the statements written in nursing homes were handwritten.

There was a large variety in the narrative content of the statements. Many were carefully written describing relevant information in a respectful way with all required data properly provided, whereas the shortest narrative text read simply "severely demented since long". The narrative text was usually written in a neutral form avoiding expressing the physician’s own contribution or opinions.

### Physician-patient relationship

The fact that a physician is responsible for a patient does not necessarily mean that they know each other. The statement contains a box for confirmation of the patient’s identity. In only 53%, the physician knew the patient, and in 7%, a relative or staff member had confirmed the identity of the patient. In addition, 5% showed a formal ID-card. In 36% of the cases, the box for confirmation of patient identity was left blank. In 4% of the cases, the physician had actually written that the patient was unknown! The identity of the patient was thus not confirmed in 40% of the cases.

### Did the patient and the physician actually meet for assessment of mental capacity?

A number of facts can easily be retrieved from the patient record, but the physician is required to meet the patient for the assessment when applying for a custodian. This is not always the case. From the narrative text, we tried to elucidate when, where and if the physician and the patient actually had met, and if they had discussed the application for a custodian. In only 20% of the statements, it was obvious that the physician actually personally had met the patient for this purpose. In 2%, it was stated that the physician did not meet the patient.

### The physician’s conclusions about patient’s mental capacity to consent, to understand and to appear in court

After writing the narrative text, the physician must answer yes or no to three questions about the patient’s mental capacity to consent, to understand and to appear in court. The answers, that are critical for the court proceedings are shown in Table 2.

**Table 2:** The physician’s answer about written consent, ability to understand and appear in court

**The physician’s answer about written consent, ability to understand and appear in court**	**Yes**	**No**	**Blank**
Did the individual give his/her written consent to the arrangement of a custodianship? If "yes", the written consent of the individual should be enclosed to the application.	40%	58%	2%
	Men 37%	Men 60%	
	Women 41%	Women 57%	
Can the individual be heard in court without causing him/her any harm?	73%	25%	2%
	Men 71%	Men 28%	
	Women 75%	Women 23%	
Is the condition of the individual such that he/she obviously does not understand what the matter concerns?	54%	44%	**2%**
	Men 56%	Men 41%	
	Women 53%	Women 45%	

Table 2 shows the physician’s answers to the questions whether the patient had given a written consent and his /her ability to understand and appear in court concerning the 260 patients aged 51–105 (Median 84) years of age. There were 87 men (Median age 79; Range 60–100) and 173 women (Median age 85: Range 51–105).

Firstly, the physician answered that 40% of the patients had signed consent, but only 89% of those were found. In another 5%, we found signed consents unknown to the physician*.* Secondly, the physicians found that 73% of the patients were able to "be heard in court without causing him/her any harm". Thirdly, in 54% of the cases the condition was "such that he/she obviously does not understand what the matter concerns". Out of this group unable to understand, the physicians found that 57% of the patients still could "be heard in court without causing him/her any harm". In 11% of those unable to understand, the physician was aware that the patient had signed consent. In another 9%, the patient unable to understand had signed consent without the physician knowing this.

### The physician’s conclusions about patient’s need for a custodian

The physician had ticked that the patient needed a custodian due to "disease" in 89%, a "weakened state of health" in 27%, "mental disorder" in 10%, and "similar condition" in 5% of the cases. Several items may be ticked and it does not influence the court procedure.

Most of the patients needed massive help with daily life, as well as economy. "Safeguard his/her rights" was ticked in 92% and "manage his/her property" in 98% and "ensure his/her personal needs" was ticked in 91%. In two cases, no item was ticked. Ticked items are relevant for the court procedure, as it influences the extent of the custodian’s appointment.

### Gender differences

There were twice as many women than men. We found no major gender differences other than, as in the general population; women were older than men and that twice as many men (8%) than women (3.4%) lived at home together with a spouse or child. Men were more often hospitalized than women at the time of assessment.

## Discussion

This study has focused on physician’s statements concerning very old, very sick, very lonely and vulnerable elderly in need of protection and assistance of others. This is a group of patients unable to complain, in 54% unable to understand, and very often without close relatives who can speak for them. One would expect that physician’s statement to be presented in court would be produced with utmost care. Just like in studies from Israel [15] and Texas [14], this was not always the case.

Why was the identity of the patients not confirmed in 40% of the cases? Did the physician not care to fill in the space for this, or did the physician write the statement extracting data from the patient record without seeing the patient? In only 20%, it was obvious that the physician actually personally had met the patient for writing this statement. It is, however, likely that this figure is too low due to the neutral way physicians write about patients, avoiding mentioning their own role. In 15 cases, the physician had ticked both that the patient was unable to understand and at the same time had signed consent, showing that the physician did not realize that a person lacking capacity should not sign or was unaware that the social worker had asked the patient to sign consent. The patient may already have been discharged when a relative or social worker requested a physician’s statement.

Advantages with the Swedish system of appointing custodians are that it is simple, cheap and close to the patient. Any licensed physician can write the statement, and the local Chief Guardians and District Courts handle the applications in close collaboration. Court proceedings are brief and lawyers are not needed. Getting a custodian appointed as soon as possible was probably more important for vast majority of patients in the study than having a perfect physician’s statement. Sweden is considered one of the least corrupted countries in the world [17]. This may contribute to a relaxed attitude when writing statements. The procedure seems relaxed also in the U.S., where the legal system is quite different from that of Sweden. In ten U.S. States, the majority of court hearings for guardianship lasted less than 15 minutes and 25% less than five minutes [18]. Medical testimony was rarely presented and only one-third of respondents were represented by an attorney during the guardianship process although the court granted 95% of guardianship requests.

We believe that bringing vulnerable elderly with memory impairment to court is usually not a good idea. Also frail elderly without diagnosed memory disorder may prefer that somebody else takes care of the court procedure. Out of 454 physician’s statements scrutinized, 194 of the statements had no mentioning of memory problems but a heavy load of other medical problems, making some memory impairment likely. Already in the group with memory impairment but not dementia, 28% were considered unable to understand, and probably suffered from dementia without having a diagnosis established.

The compulsory question in the form "Can the individual be heard in court without causing him/her any harm?" can be interpreted as two questions. The first is if the patient is able to give meaningful information to the court. The law says that a person who is unable to understand should not be heard in court in cases concerning custodian. The physician may, however, think that the patient can be taken to court so that the court can see how demented the patient is. The second question is if this would cause the patient any harm. Harm is not defined, but is likely that many patients would find a court proceeding distressing. Most court proceedings concerning custodian are done without the patient present, in spite of physicians ticking that 57% of patients unable to understand can be heard.

The majority of statements were issued by senior physicians in family practices or geriatric setting. The only physicians that wrote more than one statement in our study worked in memory clinics. Since most physicians seldom write statements to the court, they don’t get enough experience to do this well. In Australia, it has been recognized that many health professionals are unaware of the provisions of the legislation and are unclear about what information will be required [11]. Taking over decision-making for others is a multidisciplinary task, which explains some of the difficulties like physicians not understanding legal language, and courts not understand what physicians mean.

In England and Wales, the MCA [10] has been preceded by years of multidisciplinary work. Physicians, psychologists, nurses, lawyers, social workers, patient organizations and others have contributed to make a practically useful legislation. The MCA provides a modern framework to empower and protect people who may lack capacity to make some decisions for themselves. The cornerstone of the legislation is that every adult must be assumed to have decision-making capacity unless it is proved otherwise. It is adapted for many different situations in the life of a cognitively impaired person, not only for financial affairs. The MCA makes clear who can take decisions in which situations, and how they should go about this. The Code of Practice supports the MCA and provides guidance to all those who care for and/or make decisions on behalf of adults who lack capacity. There is also a specialized Court of Protection. Numerous educational activities have been made for health care professionals and others.

Given the expected increase of frail elderly and persons with dementia worldwide, there is a strong need for practically useful guidelines to ensure correct assessments and safe legal procedures. Since the courts have little medical knowledge, a practical solution for all could be to give the courts and public guardians access to specially trained physicians to help out in difficult cases. Alternatively, there could be specialized courts like in England and Wales. The MCA can serve as a model for other countries wishing to modernize their legislation and find practically useful methods of capacity assessment in different situations. The multidisciplinary approach is useful in a number of settings and not limited to the health care system. The participation of the public and patient organisations is essential in order to enable people to decide for themselves and their loved ones as much as possible.

## Conclusion

In conclusion, many physicians’ statements about the mental capacity of vulnerable elderly patients had serious short-comings. Physicians in Sweden and worldwide are often unaware of the provisions of the legislation and how to write a statements. Given the expected increase of frail elderly and persons with dementia, there is a strong need for guidelines to ensure correct assessments and safe legal procedures. Legislations differ between countries, but the problems are similar. Due to the world-wide migration, there is a need for internationally accepted concept. Development of guidelines and changes in legislations will benefit from multidisciplinary collaboration to avoid misunderstandings between the health and social care systems and the legal system. Medical schools should offer education about capacity assessment and the legal framework to protect vulnerable elderly, and professionals need continuous education in the field. Last but not least, the public must be involved.

## Competing interests

The authors declare that they have no competing interests.

## Authors’ contributions

KSB and KF conceived of the study. KSB scrutinized all files. KF contributed with expertise from the Public Guardian’s point of view and KSB and JU from the medical point of view in analysis of data. KSB drafted the manuscript, and all authors contributed with their expertise. All authors read and approved the final manuscript.

## Authors’ information

Kerstin Fälldin, Chief Guardian’s Office of the City of Stockholm and Johanna Ulfvarson, Department of Neurobiology, Care Sciences and Society, Karolinska Institutet, Stockholm, Sweden.

## Pre-publication history

The pre-publication history for this paper can be accessed here:

http://www.biomedcentral.com/1471-2318/14/94/prepub

## Supplementary Material

Additional file 1 Shows the English translation of the official form for physician’s statement for assessment in cases concerning arrangement of custodianship established by the National Board of Health and Welfare. Click here for file
