# Supplementary material for: Custodian for elderly with memory impairment in Sweden – a study of 260 physicians’ statements to the court
Source: BMC Geriatr. 2014 Aug 26;14:94. doi: 10.1186/1471-2318-14-94 (PMC4161266; doi:10.1186/1471-2318-14-94)
Supplement: Additional file 1 — Shows the English translation of the official form for physician’s statement for assessment in cases concerning arrangement of custodianship established by the National Board of Health and Welfare. [file 1471-2318-14-94-S1.pdf]

The National Board of Health and Welfare

THIS TRANSLATION IS MADE BY THE AUTHORS AND IS NOT AN OFFICIAL VERSION.

PHYSICIAN´S STATEMENT

for assessment in cases concering arrangement of custodianship (“Good Man”) according to the Children and Parents Code[1] (Föräldrabalken); 11th Chapter 4§

”If someone, due to disease, mental disorder, weakened state of health or similar condition needs help to safeguard his/her rights, to manage his/her property or to ensure his/her personal needs, the court should, if so required, decide to arrange a custodianship for him or her. This may not be decided without the consent of the person for whom the custodianship is to be arranged, unless the condition of the individual prevents that his/her opinion is obtained.

When the court pronounces a decision according to the first paragraph, the court shall at the same time appoint a custodian to execute the mission. If a custodian in any other case should be appointed on the basis of the first paragraph, the appointment shall be prounced by the Chief Guardian.”

Reset

Personal data about the person that the statement refers to

Last name and first name		Personal identity number
Street address	Postal code	City
Occupation/title		Phone (including area code)
Site of civil registration		Identity verified by

Basis for assessment

Current state of health

1 Additional provisions are found in the Children and Parents Code (Föräldrabalken) 11th Chapten 17§; ”Before the court arranges a trusteeship, a physician´s statement or an equivalent assessment about the health condition of the individual shall be acquired. This also applies in cases concerning the arrangement of a custodian according to §4, when a consent of the individual was not obtained”

Last name and first name	Personal identity number

Basis for assessment (cont´d)

Circumstances which suggest that the individual needs to help to safeguard his/her rights, to manage his/her property or to ensure his/her personal needs

Prior to proceedings in the District Court

Did the individual give his/her written consent to the arrangement of a custodianship?<br>If "yes", the written consent of the individual should be enclosed to the application.	☐ Yes	☐ No
Can the individual be heard in court without causing him/her any harm?	☐ Yes	☐ No
Is the condition of the individual such that he/she obviously does not understand what the matter concerns?[2]	☐ Yes	☐ No

Statement

I hereby certify that I, after an assessment based on a personal examination of the patient and with the support of other information, have found that

………

(the name of the individual)

Needs help to	☐ safeguard his/her right	Because of	☐ disease
	☐ manage his/her property		☐ mental disorder
	☐ ensure his/her personal needs		☐ weakened state of health
			☐ similar condition

Signature

Place and date	Signature of the licensed physician

Last name and first name of the licensed physician	Position

Workplace

Street address	Postal code	City

Phone (including area code)	Telefax number	E-mail address

2 What you have to assess is whether the individual because of his/her condition obviously does not understand what the matter concerns. If this is your judgement, the question shall be answered "yes".
